# Diagnosis and management of polycystic ovary syndrome in the UK (2004–2014): a retrospective cohort study

**DOI:** 10.1136/bmjopen-2016-012461

**Published:** 2016-07-11

**Authors:** Tao Ding, Gianluca Baio, Paul J Hardiman, Irene Petersen, Cormac Sammon

**Affiliations:** 1 Department of Statistical Science, University College London, London, UK; 2 Institute for Women's Health, University College London Medical School, London, UK; 3 Department of Primary Care and Population Health, University College London, London, UK

**Keywords:** EPIDEMIOLOGY, Polycystic Ovary Syndrome, prescription

## Abstract

**Objective:**

To estimate the incidence and prevalence of polycystic ovary syndrome (PCOS) in UK primary care and investigate prescribing patterns before and after a PCOS diagnosis.

**Design:**

Retrospective cohort study.

**Setting:**

UK primary care (2004–2014).

**Participants:**

Women aged 15–45 years.

**Primary and secondary outcome measures:**

The incidence and prevalence of diagnosed PCOS and probable PCOS (ie, those without a confirmed diagnosis but with at least 2 PCOS features recorded within 3 years). Among women with diagnosed or probable PCOS, the prevalence of prescribing of drugs typically used to treat PCOS was calculated prior to and in the 24 months after the diagnosis of PCOS.

**Results:**

We identified 7233 women with PCOS diagnoses and 7057 women with records suggestive of probable PCOS, corresponding to incidence rates of 0.93 and 0.91 per 1000 person-years at risk (PYAR) and an overall rate of 1.84 per 1000 PYAR. Women aged 20–24 years and women living in deprived areas had the highest incidence of PCOS. The prevalence of PCOS in 2014 was ∼2%. The proportion of women with a prescription in the 24 months after their PCOS index date varied by drug type: 10.2% metformin, 15.2% combined oral contraceptives, 18.8% acne-related treatments, 1.93% clomiphene, 1.0% spironolactone, 0.28% cyproterone and 3.11% eflornithine. Acne-related treatments were more commonly used to treat probable (28.3%) than diagnosed (12.3%) cases, while metformin was prescribed much more commonly in diagnosed cases.

**Conclusions:**

In conclusion, compared to rates estimated in community samples, the incidence and prevalence of women presenting in primary care with PCOS diagnoses and features are low, indicating that PCOS is an under-recognised condition. Although considerable variation is observed in treatments prescribed to women with PCOS, the treatments initiated following a confirmed diagnosis generally reflect the long-term prognostic concerns raised in PCOS consensuses.

**Strengths and limitations of this study**

- The current study is the first to investigate the incidence and prevalence of polycystic ovary syndrome (PCOS) in the primary care setting in the UK and the longitudinal nature of the database allowed us to examine trends over a long time period, which has not been captured by previous epidemiological studies.
- Underdiagnosis was the main concern for the current study as only data considered relevant at the time of a consultation are recorded by clinicians although we attempted to include women with two or more features of PCOS as potential cases.
- Our study investigated the prescribing patterns of PCOS in the UK primary care, which has not been well explored in previous studies. These findings reflected the current management of PCOS in the clinical practices and provide important indications for general practitioners.

## Background

Polycystic ovary syndrome (PCOS) is associated with a wide range of reproductive, cardiometabolic and dermatological abnormalities. One of the most prominent symptoms in patients with PCOS is oligomenorrhea. Consequently, women with PCOS are highly likely to be infertile and potentially develop endometrial hyperplasia due to continuing secretion of oestrogen without ovulation.^1^ ^2^ Furthermore, emerging evidence has suggested that ∼50–70% of patients with PCOS have insulin resistance regardless of their body weight or body mass index.^3^ Consequently, women with PCOS are at an elevated risk of developing various common metabolic disorders compared with the general population.^4^ In addition, many patients with PCOS are observed to have an elevated androgen level, which leads to hirsutism, alopecia and acne.^1^

While the epidemiology of PCOS in the community has been well studied,^5–8^ the proportion of women who present in routine clinical practice with PCOS features and the extent to which these women are subsequently diagnosed are less clear. Similarly, while a range of treatments have been suggested for the management of PCOS,^9^ there is very little information regarding which of these drugs are actually prescribed in routine clinical practice. Such ‘real-world evidence’ can help identify priority areas for research, training and health promotion efforts. The current study sought to provide such evidence by investigating the recording of PCOS features and diagnoses in UK general practice between 2004 and 2014 and the subsequent prescribing of pharmacological treatments.

## Methods

### Data source

The Health Improvement Network (THIN) is one of the largest primary care data sources in the UK, including data from over 500 general practices, covering ∼6.2% of the total population in the UK. Available data include patient demographics, medical history, test results, drug prescriptions and social deprivation as measured by quintiles of the Townsend score.^10^ Symptoms and diagnoses are recorded using a hierarchical clinical coding system (Read codes),^11^ with additional information recorded as unstructured text. The information stored as unstructured text was not available in this study. Notably, as the data are collected in routine clinical practice, only information deemed clinically relevant is entered in a patient's record.

In our study, data were included from each practice that met minimum quality criteria, for example, acceptable computer usage (a time point when a practice is considered to use their computer system adequately, ie, at least one medical record, one additional health data record such as body mass index, laboratory test results and two therapy records are computerised annually for a practice) and acceptable mortality reporting (a time point which the observed death rates for a practice reach the standard predicted numbers of deaths derived from National statistics given the practice's demographics).^12–14^

### Study population

Women aged 15–45 years, who were permanently registered for at least 1 year, were included in the study population. Women with conditions that can cause similar symptoms to PCOS were identified and excluded. These conditions include prolactinoma, Cushing's syndrome, Nelson's syndrome, adrenal-related disorders (ie, adrenal tumours, adrenal hyperplasia) and pituitary disorders.

### Case definition

PCOS cases were identified using two methods. First, Read codes for ‘polycystic ovary syndrome’ (C165.00), ‘Stein-Leventhal syndrome’ (C164.12) and ‘endoscopic drilling of ovary’ (7E25300) were used to identify those women who had been clinically diagnosed as PCOS cases (diagnosed cases). Women with two or more Read codes indicative of PCOS features (menstrual/ovarian dysfunction, clinical and biochemical hyperandrogenism, polycystic ovaries) recorded in a 3-year period were then selected and we considered these as probable cases. These women were considered as those who were likely to meet at least one of the three major definitions of PCOS^15–18^ but who may not have been clinically diagnosed as having the condition. The index date for probable cases was considered to be the date the second PCOS feature was recorded. A full list of the codes used to define cases is provided in online supplementary table SI.

10.1136/bmjopen-2016-012461.supp1 Supplementary data

### Covariates and prescription indicators

We extracted data on each woman's year of birth, ethnicity and deprivation level of the area in which the woman lived;^10^ data on prescriptions of interest (ie, combined oral contraceptives (COCs), progestin oral contraceptives (POCs), intrauterine devices, clomiphene, metformin, spironolactone, gonadotrophins, cyproterone, flutamide, eflornithine, weight control/loss drugs, lipid regulators and acne-related drugs) were also included and information on prescribing of these drugs before and in the 24 months after each PCOS case index data was extracted.

### Statistical analysis

For incidence estimation, the rate was computed as the total number of new PCOS cases recorded between 2004 and 2014 divided by the total number of person-years of follow-up. Person-time for the denominator was estimated by summing each woman's follow-up from the latest among (1) their 15th birthday, (2) 1 year after registration, (3) the date at which their practice met minimum quality criteria and (4) the 1 January 2004, to the earliest of the date among (1) their first incident diagnosis, (2) their date of death, (3) the date they left the practice, (4) the date data were last collected from their practice and (5) the 31 December 2014. All incidence rates were reported per 1000 person-years at risk (PYAR).

Hierarchical (patients were considered to be nested in each practice) multivariate Poisson regression models were used to estimate incidence rate ratios and 95% CIs comparing the incidence of first PCOS diagnoses across 5-year age bands, Townsend score quintiles and calendar period (ie, 2004–2007, 2008–2011 and 2012–2014).

The period prevalence of the diagnosis of PCOS was evaluated for the calendar year 2014. The denominator for the prevalence calculation consisted of any women with at least 1 year of postregistration follow-up, of which at least 6 months must have occurred in 2014. The prevalence of PCOS was also estimated within 5-year age bands. Secondary analysis was carried out to assess the sensitivity of the prevalence estimate to the length of the postregistration period (ie, 1 year, 2 years) and the minimum period registered within 2014 (ie, 3, 6 and 9 months).

Among the women with a diagnosis of PCOS, we calculated the number and proportion with a prescription for one of the drugs of interest at any point prior to their PCOS index date. Among the women without prescription for each drug of interest prior to the index date, we then calculated the proportion of women with a prescription for that drug within 2 years after the PCOS index date. We used cumulative incidence plots to describe how the proportion initiating different drugs increased over the 2 years following the PCOS index date for the time period 2004–2012.

All analyses were performed using STATA V.13.0 and were carried out for all PCOS cases and stratified by case definition (diagnosed PCOS vs probable PCOS).

## Results

In total, 7233 diagnosed and 7057 probable PCOS cases were identified among 2 087 107 female individuals aged 15–45 years old between 2004 and 2014. Table 1 describes the number of PCOS features identified in each group. The incidence rate of diagnosed PCOS cases was 0.93 per 1000 PYAR (95% CI 0.91 to 0.96), whereas the rate for probable cases was 0.91 per 1000 PYAR (95% CI 0.89 to 0.93). This equated to an overall combined incidence rate of 1.84 PYAR (95% CI 1.81 to 1.87).

**Table 1:** Number and proportion of diagnosed and probable cases with major PCOS features

Features	Diagnosed cases (n=7233)	Probable cases (n=7057)
Menstrual dysfunction	2055 (28.4)	6265 (88.8)
Hyperandrogenism	2836 (39.2)	6221 (88.2)
PCO	199 (2.8)	1636 (23.2)
Two or more features	597 (8.3)	7057 (100)

Values are represented as n (%).

PCOS, polycystic ovary syndrome.

**Figure 1:**
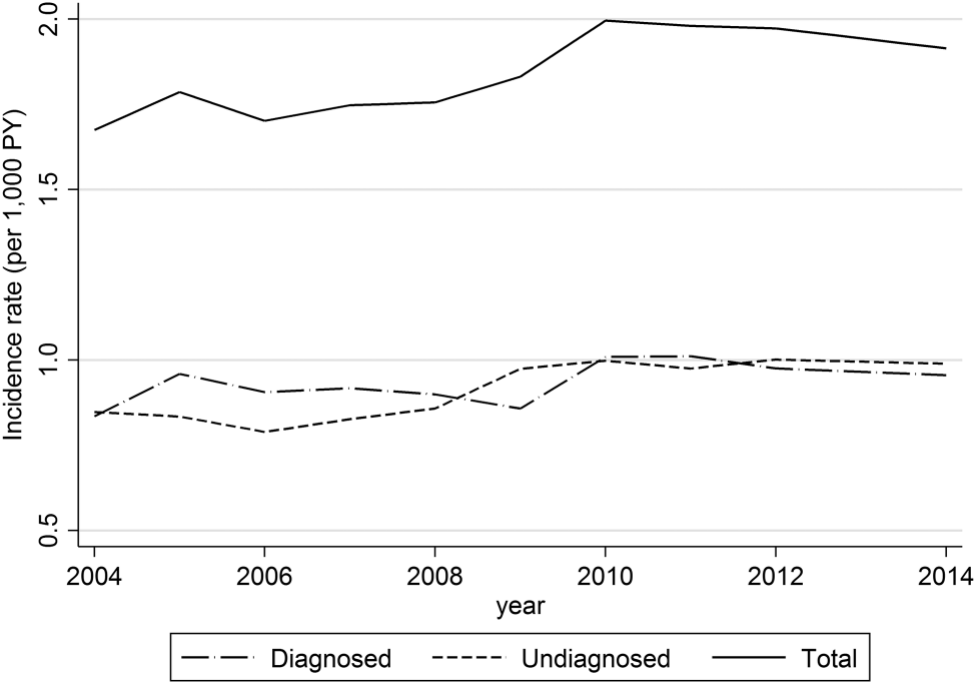
Time trends in PCOS diagnosis recorded (for diagnosed, probable and total cases). PCOS, polycystic ovary syndrome.

The overall incidence of PCOS increased from 1.67 (95% CI 1.58 to 1.77) per 1000 PYAR in 2004 to 2.00 (95% CI 1.89 to 2.10) per 1000 PYAR in 2010, after which the rate remained relatively constant at ∼2 per 1000 PYAR (figure 1). The incidence was the highest for those in the 20–24 year age group (3.59 per 1000 PYAR, 95% CI 3.47 to 3.70), whereas the 40–44 year age group had the lowest incidence (0.62 per 1000 PYAR, 95% CI 0.58 to 0.66). The age-specific trend of PCOS diagnoses was similar for diagnosed and probable cases. After adjusting for the effects of year and social deprivation, significant differences still remained in the incidence of PCOS (table 2). In terms of social deprivation, the incidence of PCOS for individuals who were least deprived was 1.59 (95% CI 1.53 to 1.65) per 1000 PYAR, whereas among the most deprived, a rate of 2.23 (95% CI 2.15 to 2.32) per 1000 PYAR was estimated. This difference in rates remained statistically significant after adjusting for effects of other covariates (ie, age and year) and after stratifying by case definition (table 2).

**Table 2:** Recorded rate of PCOS diagnoses by social and demographical characteristics

	Diagnosed PCOS			Probable PCOS			Overall		
	Rate per 1000 PYAR (95% CI)	Adjusted* IRR (95% CI)	p Value	Rate per 1000 PYAR (95% CI)	Adjusted* IRR (95% CI)	p Value	Rate per 1000 PYAR (95% CI)	Adjusted* IRR (95% CI)	p Value
Townsend quintile			<0.001			<0.001			<0.001
1	0.80 (0.76 to 0.84)	1		0.80 (0.76 to 0.84)	1		1.59 (1.53 to 1.65)	1	
2	0.90 (0.85 to 0.95)	1.14 (1.06 to 1.23)		0.79 (0.75 to 0.84)	0.99 (0.92 to 1.07)		1.69 (1.62 to 1.75)	1.08 (1.02 to 1.14)	
3	0.94 (0.90 to 0.99)	1.12 (1.04 to 1.21)		0.91 (0.86 to 0.96)	1.07 (0.99 to 1.15)		1.85 (1.78 to 1.92)	1.10 (1.05 to 1.16)	
4	1.02 (0.97 to 1.07)	1.15 (1.06 to 1.24)		0.97 (0.92 to 1.02)	1.06 (0.98 to 1.15)		1.98 (1.91 to 2.05)	1.11 (1.05 to 1.17)	
5	1.07 (1.01 to 1.13)	1.15 (1.05 to 1.25)		1.17 (1.11 to 1.23)	1.21 (1.11 to 1.32)		2.23 (2.15 to 2.32)	1.19 (1.11 to 1.26)	
Age (years)			<0.001			<0.001			<0.001
15–19	1.20 (1.14 to 1.27)	0.69 (0.64 to 0.74)		0.54 (0.50 to 0.59)	0.29 (0.27 to 0.32)		1.75 (1.67 to 1.83)	0.49 (0.46 to 0.51)	
20–24	1.72 (1.64 to 1.80)	1		1.87 (1.79 to 1.96)	1		3.59 (3.47 to 3.70)	1	
25–29	1.51 (1.44 to 1.58)	0.86 (0.80 to 0.91)		1.49 (1.42 to 1.56)	0.78 (0.73 to 0.83)		2.98 (2.88 to 3.08)	0.81 (0.78 to 0.85)	
30–34	1.03 (0.98 to 1.09)	0.58 (0.54 to 0.62)		0.86 (0.81 to 0.91)	0.46 (0.42 to 0.49)		1.88 (1.81 to 1.96)	0.51 (0.49 to 0.54)	
35–39	0.45 (0.42 to 0.49)	0.26 (0.24 to 0.28)		0.55 (0.51 to 0.59)	0.30 (0.27 to 0.32)		0.99 (0.94 to 1.05)	0.27 (0.26 to 0.29)	
40–44	0.17 (0.15 to 0.19)	0.10 (0.08 to 0.11)		0.45 (0.42 to 0.48)	0.24 (0.22 to 0.26)		0.62 (0.58 to 0.66)	0.17 (0.16 to 0.18)	
Year			<0.001			<0.001			<0.001
2004–2007	0.91 (0.87 to 0.94)	1		0.82 (0.79 to 0.86)	1		1.73 (1.68 to 1.78)	1	
2008–2011	0.94 (0.91 to 0.98)	1.00 (0.95 to 1.06)		0.95 (0.92 to 0.99)	1.13 (1.07 to 1.19)		1.89 (1.84 to 1.94)	1.06 (1.02 to 1.10)	
2012–2014	0.96 (0.92 to 1.01)	0.98 (0.92 to 1.04)		0.98 (0.93 to 1.02)	1.13 (1.07 to 1.20)		1.92 (1.86 to 1.98)	1.04 (1.00 to 1.09)	

*IRR from multilevel Poisson distribution accounted for practice-level variability and adjusted for other variables considered.

IRR, incidence rate ratio; PCOS, polycystic ovary syndrome; PYAR, person-years at risk.

The overall prevalence of PCOS in 2014 was ∼2.27% (95% CI 2.23% to 2.31%), with a prevalence of 1.34% and 0.93% in diagnosed and probable cases, respectively. The age-specific prevalence peaked in the 30–34 year age group, and decreased for older age groups. Prevalence estimates were not sensitive to varying the postregistration period and the time period registered within 2014, remaining consistently ∼2%.

The proportion of women using one of the PCOS-related drugs before or after their index date varied widely across drugs groups (see table 3). At the time of their PCOS index date, over 40% of women had previously been prescribed COC, ∼30% had been prescribed acne-related drugs before diagnosis, >18% had been prescribed POCs and ∼18% had previously been prescribed at least one of the other drugs (table 3). Acne-related drugs, COC and metformin were the most commonly used drugs in the 24 months after a PCOS record (table 3). Plots describing the cumulative incidence of women with a prescription for each drug type over the 24 months following their index date are provided in online supplementary figure SI. The plots show that while all drugs show an initial surge in prescribing on or just after the PCOS index date, this is greater for some drugs (eg, metformin, acne-related drugs) than for others (COCs and POCs).

**Table 3:** Number and percentage of PCOS women prescribed relevant drugs for PCOS prior to and following the diagnosis of PCOS

Types of drugs	Before diagnosis of PCOS, number (%)	After diagnosis of PCOS, number (%)		
		2004–2007	2008–2011	2012–2014
Combined oral contraceptives	12 349 (40.22)	901 (17.01)	912 (18.87)	459 (17.88)
POC	5806 (18.91)	474 (6.45)	691 (10.22)	272 (7.80)
IUDs	728 (2.37)	43 (0.50)	64 (0.75)	25 (0.52)
Clomiphene	518 (1.69)	210 (2.46)	165 (1.92)	70 (1.43)
Metformin	1278 (4.16)	1212 (14.77)	1102 (13.25)	495 (10.52)
Gonadotrophins	435 (1.42)	29 (0.34)	13 (0.15)	13 (0.26)
Spironolactone	235 (0.77)	109 (1.26)	111 (1.28)	43 (0.87)
Cyproterone	91 (0.30)	28 (0.32)	19 (0.22)	3 (0.06)
Flutamide	6 (0.02)	4 (0.05)	1 (0.01)	0
Eflornithine	550 (1.79)	354 (4.10)	407 (4.81)	172 (3.58)
Weight control/loss drugs	1330 (4.33)	403 (4.84)	364 (4.43)	91 (1.95)
Lipid regulators	194 (0.63)	69 (0.79)	46 (0.53)	8 (0.16)
Acne-related drugs	9000 (29.31)	1182 (19.52)	1234 (20.78)	582 (18.32)

Values are represented as n (%).

COC, combined oral contraceptive; IUD, intrauterine device; PCOS, polycystic ovary syndrome; POC, progestin oral contraceptive.

**Figure 2:**
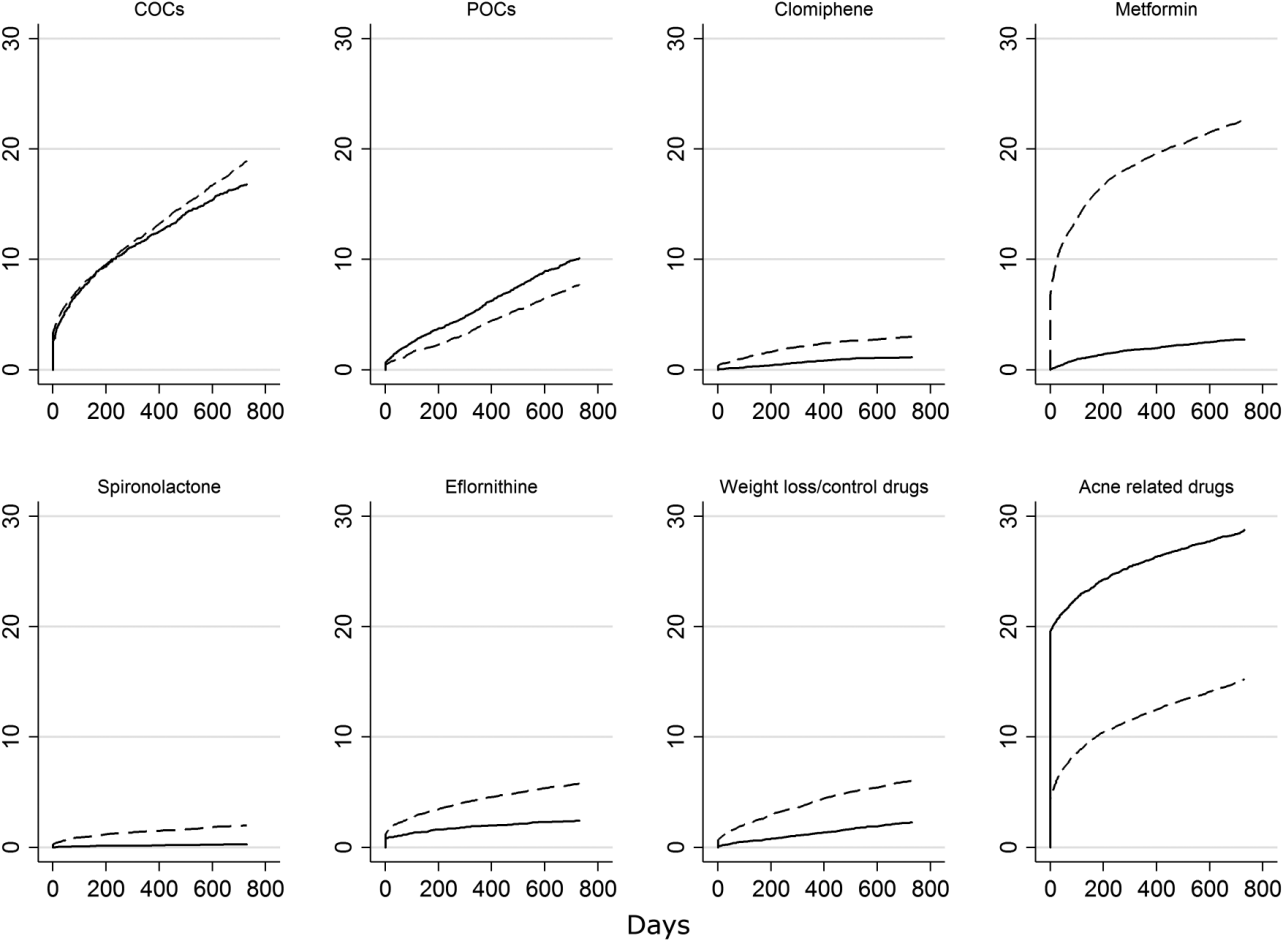
Plots describing the cumulative incidence of women with a prescription for each drug type over the 24 months following their index date stratified according to whether the case was diagnosed (dashed line) or probable (solid line). Results shown for the eight most commonly prescribed drugs. COC, combined oral contraceptive; POC, progestin oral contraceptive.

Prescription results stratified according to whether PCOS cases were diagnosed or probable are provided in online supplementary table SII. These results indicate that acne-related treatments and POCs were more commonly used to treat probable than diagnosed cases, while COCs, metformin, clomiphene, cyproterone, eflornithine and weight loss drugs were prescribed more commonly in diagnosed than probable cases. Cumulative incidence plots stratified according to whether a case was diagnosed or probable illustrate the differences listed in online supplementary table SII and further to this show that these differences are typically established on or immediately after the index date (figure 2).

## Discussion

### Summary

We present data on >14 000 potential PCOS cases among women aged 15–45 years in primary care across the UK between 2004 and 2014. 51.2% of these women had a PCOS diagnosis recorded, while 49.9% did not, corresponding to incidence rates of 0.93 per 1000 PYAR (95% CI 0.91 to 0.96) and 0.91 per 1000 PYAR (95% CI 0.89 to 0.93), respectively. The prevalence of PCOS in 2014 was ∼2.27%. There was a considerable variation in the type of drug prescribed on the day of, or in the 24 months after, a PCOS diagnosis and prescribing differed between diagnosed and probable cases.

### Strengths and limitations

To the best of our knowledge, this study is the first to investigate the diagnosis and management of PCOS in the primary care setting in the UK. As THIN contains over 10 million patient records, our study is robust in terms of sample size. The longitudinal nature of the database also allowed us to examine trends over a 10-year study period, which has not been captured by previous epidemiological studies where individuals were often sampled at a single time point.

As our data were collected in routine clinical practice, our results reflect the true burden of PCOS on the healthcare system. However, this also means that only data considered relevant at the time of a consultation are recorded by clinicians. Consequently, it is unsurprising that only 8% of diagnosed cases had two or more PCOS features recorded as, while the initial feature prompting referral is likely to be noted by the general practitioner (GP), once a PCOS diagnosis has been made by a specialist a GP is unlikely to record anything other than the confirmed diagnosis in the coded record. The routine nature of data collection also meant that underdiagnosis was a concern in the current study and underestimation of PCOS rates was anticipated. We attempted to address under-reporting by allowing women with two or more features of PCOS (interfeature period within 3 years) to count as a PCOS case. However, the inclusion of probable cases may introduce case misclassification as some probable cases may not be true PCOS cases. For example, while we considered women with a raised testosterone level to have hyperandrogenism, there are concerns surrounding the accuracy of testosterone testing.^19^ On the contrary, it is also possible that many probable cases are true cases but do not have a diagnosis recorded in their medical records for some reason.

Incomplete patient history is a concern as women with prevalent PCOS diagnoses at the time of registration with a practice may not be identified, resulting in the underestimation of prevalence rates and the overestimation of incidence rates. Additionally, the lack of information on ethnicity is also an issue as the trends in incidence observed over age, deprivation and calendar year categories may be influenced by unobserved differences in ethnicity distributions across these covariates.

As we lack information on the indication for prescriptions, we cannot be certain that prescriptions issued after, or even on, the date of a PCOS record were prescribed for the treatment of PCOS. For example, ∼30% of the PCOS cases prescribed metformin had a prior diagnosis of type 2 diabetes, the approved indication for this drug. However, by excluding those ever prescribed metformin prior to their PCOS diagnosis from our calculations, we can be relatively confident that prescriptions for metformin issued on the date of a PCOS diagnosis are likely to be at least partly for the treatment of PCOS. Our confidence that the drug was prescribed for PCOS then decreases with increasing time after the PCOS diagnosis such that we expect the proportion of diagnosed cases prescribed metformin for PCOS to lie somewhere between the 8% with a prescription on the date of diagnosis and the 20% with a prescription on or in the 24 months after the date of diagnosis.

While we lacked information on prescribing outside primary care, the responsibility for prescribing treatments which have been initiated by specialists is likely to be transferred to an individual's GP a number of months after diagnosis. The use of a 24-month window to assess prescribing therefore allowed us to capture initiation of drugs prescribed in secondary care. This is reflected in the cumulative incidence curves shown in online supplementary figure S1 where the drugs that are commonly initiated outside primary care (eg, spironolactone and clomiphene) are initiated further after the index date than the drugs typically initiated in primary care (eg, COCs and acne drugs). However, prescribing rates may be underestimated if care is not transferred to the GP within 24 months (eg, for budgetary reasons).

The proportion of women with PCOS who had been prescribed COCs before their PCOS diagnosis is similar to that reported by other studies.^20^ ^21^ The proportion of women with PCOS who initiated metformin after their diagnosis in our study (over 10%) is comparable to that of a Danish study where 11.8% of the women with PCOS were identified as having received metformin.^21^ These comparisons support the validity of our prescribing data.

### Interpretation

The prevalence of recorded PCOS in UK primary care in 2014 is comparable to that obtained from studies using databases in the USA (0.56–2.22%).^22–24^ However, the rates are significantly lower than those from epidemiological surveys in the Europe where systematic screening was often provided to identify cases from selected populations.^25–28^ This gap highlights the importance for improving public and GPs' awareness of PCOS.

The incidence of PCOS increased slightly over the study period; however, no significant changes in yearly rates were observed. This might reflect the increasing awareness of the syndrome after the establishment of the Rotterdam and Androgen Excess Society criteria during the study period. However, it could also be due to the improvement of the database, that is, the completeness of medical recordings has improved over time.

It should be noted that the Townsend score represents the deprivation level of the area in which a woman lives. Women who lived in more deprived areas had a higher incidence of PCOS than those living in the less deprived areas. A possible explanation is that obesity (a factor strongly associated with PCOS) is more prevalent among women living in more deprived areas. Alternatively, these women may consult their GP more frequently than those in less deprived areas, for other morbidities (ie, type 2 diabetes), and therefore have more opportunity for PCOS to be diagnosed and recorded.

The fact that the incidence of probable PCOS cases was as high as the incidence of diagnosed cases indicates that there is a large group of women who present in primary care exhibiting two features of PCOS within a 3-year period but who do not have a subsequent PCOS diagnosis. While for some of these probable cases a PCOS diagnosis may not be relevant, it is likely that a considerable proportion of the women may meet the diagnostic criteria for PCOS and should therefore be referred for further assessment. Failure to refer such women may mean that they are not offered the lifestyle advice or medications that could reduce their risk of long-term PCOS-related complications.

Variation was observed in the treatments prescribed to diagnosed and probable PCOS cases; in particular, a greater proportion of diagnosed women received metformin prescriptions, while a greater proportion of the probable cases received treatment for the PCOS feature they presented with. This suggests that the diagnosed and probable cases are indeed receiving different care for their condition, with some probable cases not receiving potentially effective treatments such as metformin. The wide variation in prescribing patterns may also be due to the varied nature of clinical presentations of PCOS not only by individuals and also by age. For example, young women consulting their GPs are more likely to ask for drugs to regulate their menses or to treat acne, whereas more elderly women may initiate antidiabetic drugs to prevent rapid conversion to diabetes.

Metformin and oral contraceptives were the two drugs most commonly initiated in women with diagnosed PCOS, possibly reflecting the major concerns of long-term metabolic risks of this syndrome stated by the three PCOS consensuses. However, it is notable that even among the diagnosed PCOS cases, there is some variation in treatments prescribed following a diagnosis. This suggests that there may be a lack of consensus on the ideal treatment for the condition. This is supported by a recent survey of European endocrinologists which found variation in the treatments most commonly prescribed for PCOS.^29^ Further research into the comparative efficacy and effectiveness of the various PCOS treatment options may therefore be warranted.

### Conclusions

In conclusion, compared to rates estimated in community samples, the incidence of women presenting in primary care with PCOS diagnoses and features is low compared with most epidemiological surveys. Among the women who present, only 50% were observed to have a PCOS diagnosis recorded. Further work is therefore needed to inform women and healthcare professionals about the condition to avoid any worsening of the disease or rapid conversion into other metabolic disorders considering the relatively low cost of diagnosis and high cost of care for the associated diseases suggested by Azziz *et al*.^30^ There is much potential for these treatments to prove cost-effective alternatives, which should be carefully considered by public healthcare providers, such as the National Health Service in the UK.

Although there is much variation in the treatments prescribed following a PCOS diagnosis, the widespread prescribing of oral contraceptives and metformin generally reflects the prognostic concerns raised in PCOS consensuses, aiming to reduce the future metabolic risks of patients with PCOS or patients who are undergoing treatment for PCOS and may already have developed metabolic disorders. Further work is needed to identify the most effective treatment for the condition.

## References

[1] Balen AH, Conway GS, Homburg R Polycystic ovary syndrome: a guide to clinical management. UK: Taylor & Francis, 2005.

[2] Cheung AP Ultrasound and menstrual history in predicting endometrial hyperplasia in polycystic ovary syndrome. Obstet Gynecol 2001;98:325–31. 11506853 10.1016/s0029-7844(01)01432-6

[3] Dunaif A Insulin resistance and the polycystic ovary syndrome: mechanism and implications for pathogenesis. Endocr Rev 1997;18:774–800. 9408743 10.1210/edrv.18.6.0318

[4] Moran LJ, Misso ML, Wild RA Impaired glucose tolerance, type 2 diabetes and metabolic syndrome in polycystic ovary syndrome: a systematic review and meta-analysis. Hum Reprod Update 2010;16:347–63. 10.1093/humupd/dmq001 20159883

[5] Azziz R, Woods KS, Reyna R The prevalence and features of the polycystic ovary syndrome in an unselected population. J Clin Endocrinol Metab 2004;89:2745–9. 10.1210/jc.2003-032046 15181052

[6] Sanchón R, Gambineri A, Alpañés M Prevalence of functional disorders of androgen excess in unselected premenopausal women: a study in blood donors. Hum Reprod 2012;27:1209–16. 10.1093/humrep/des028 22343706

[7] Li R, Zhang Q, Yang D Prevalence of polycystic ovary syndrome in women in China: a large community-based study. Hum Reprod 2013;28:2562–9. 10.1093/humrep/det262 23814096

[8] Joham AE, Ranasinha S, Zoungas S Gestational diabetes and type 2 diabetes in reproductive-aged women with polycystic ovary syndrome. J Clin Endocrinol Metab 2014;99:E447–452. 10.1210/jc.2013-2007 24081730

[9] Badawy A, Elnashar A Treatment options for polycystic ovary syndrome. Int J Womens Health 2011;3:25–35. 10.2147/IJWH.S11304 21339935 PMC3039006

[10] Townsend P, Phillimore P, Beattie A Health and deprivation: inequalities and the north. London: Croom Helm, 1988.

[11] Chisholm J The Read clinical classification. BMJ 1990;300:1092 10.1136/bmj.300.6732.1092 2344534 PMC1662793

[12] Horsfall L, Walters K, Petersen I Identifying periods of acceptable computer usage in primary care research databases. Pharmacoepidemiol Drug Saf 2013;22:64–9. 10.1002/pds.3368 23124958

[13] Maguire A, Blak B, Thompson M The importance of defining periods of complete mortality reporting for research using automated data from primary care. Pharmacoepidemiol Drug Saf 2009;18:76–83. 19065600 10.1002/pds.1688

[14] http://csdmruk.cegedim.com/our-data/data-quality.html (accessed 10 Oct 2013).

[15] Zawadzki JK, Dunaif A Diagnostic criteria for polycystic ovary syndrome: towards a rational approach. In: Dunaif A, Givens JR, Haseltine FP, eds Polycystic ovary syndrome. Boston: Blackwell Scientific Publications, 1992:377–84.

[16] Rotterdam ESHRE/ASRM-Sponsored PCOS Consensus Workshop Group. Revised 2003 consensus on diagnostic criteria and long-term health risks related to polycystic ovary syndrome. Fertil Steril 2004;81:19–25. 10.1016/j.fertnstert.2003.10.004 14711538

[17] Rotterdam ESHRE/ASRM-Sponsored PCOS consensus workshop group. Revised 2003 consensus on diagnostic criteria and long-term health risks related to polycystic ovary syndrome (PCOS). Hum Reprod 2004;19:41–7. 10.1093/humrep/deh098 14688154

[18] Azziz R, Carmina E, Dewailly D Positions statement: criteria for defining polycystic ovary syndrome as a predominantly hyperandrogenic syndrome: an Androgen Excess Society guideline. J Clin Endocrinol Metab 2006;91:4237–45. 16940456 10.1210/jc.2006-0178

[19] Goodman NF, Cobin RH, Futterweit W American Association of Clinical Endocrinologists, American College of Endocrinology, and Androgen Excess and PCOS Society disease state clinical review: guide to the best practices in the evaluation and treatment of polycystic ovary syndrome—Part 1. Endocr Pract 2015;21:1291–300. 10.4158/EP15748.DSC 26509855

[20] Lo JC, Feigenbaum SL, Yang J Epidemiology and adverse cardiovascular risk profile of diagnosed polycystic ovary syndrome. J Clin Endocrinol Metab 2006;91:1357–63. 10.1210/jc.2005-2430 16434451

[21] Glintborg D, Hass Rubin K, Nybo M Morbidity and medicine prescriptions in a nationwide Danish population of patients diagnosed with polycystic ovary syndrome. Eur J Endocrinol 2015;172:627–38. 10.1530/EJE-14-1108 25656495

[22] Okoroh EM, Hooper WC, Atrash HK Prevalence of polycystic ovary syndrome among the privately insured, United States, 2003–2008. Am J Obstet Gynecol 2012;207:299.e1–7. 10.1016/j.ajog.2012.07.023 22921097

[23] Christensen SB, Black MH, Smith N Prevalence of polycystic ovary syndrome in adolescents. Fertil Steril 2013;100:470–7. 10.1016/j.fertnstert.2013.04.001 23756098 PMC3813299

[24] Sirmans SM, Parish RC, Blake S Epidemiology and comorbidities of polycystic ovary syndrome in an indigent population. J Investig Med 2014;62:868–74. 10.1097/01.JIM.0000446834.90599.5d 24844662

[25] Michelmore KF, Balen AH, Dunger DB Polycystic ovaries and associated clinical and biochemical features in young women. Clin Endocrinol (Oxf) 1999;51:779–86. 10.1046/j.1365-2265.1999.00886.x 10619984

[26] Asunción M, Calvo RM, San Millán JL A prospective study of the prevalence of the polycystic ovary syndrome in unselected Caucasian women from Spain. J Clin Endocrinol Metab 2000;85:2434–8. 10902790 10.1210/jcem.85.7.6682

[27] Diamanti-Kandarakis E, Kouli CR, Bergiele AT A survey of the polycystic ovary syndrome in the Greek island of Lesbos: hormonal and metabolic profile. J Clin Endocrinol Metab 1999;84:4006–11. 10.1210/jcem.84.11.6148 10566641

[28] Lauritsen MP, Bentzen JG, Pinborg A The prevalence of polycystic ovary syndrome in a normal population according to the Rotterdam criteria versus revised criteria including anti-Mullerian hormone. Hum Reprod 2014;29:791–801. 24435776 10.1093/humrep/det469

[29] Conway G, Dewailly D, Diamanti-Kandarakis E ESE PCOS Special Interest Group. European survey of diagnosis and management of the polycystic ovary syndrome: results of the ESE PCOS Special Interest Group's Questionnaire. Eur J Endocrinol 2014;171:489–98. 10.1530/EJE-14-0252 25049203

[30] Azziz R, Marin C, Hoq L Health care-related economic burden of the polycystic ovary syndrome during the reproductive life span. J Clin Endocrinol Metab 2005;90:4650–8. 15944216 10.1210/jc.2005-0628

