# Supplementary material for: Diagnosis and management of polycystic ovary syndrome in the UK (2004–2014): a retrospective cohort study
Source: BMJ Open. 2016 Jul 11;6(7):e012461. doi: 10.1136/bmjopen-2016-012461 (PMC4947736; doi:10.1136/bmjopen-2016-012461)
Supplement: Supplementary data [file bmjopen-2016-012461supp.pdf]

Data supplement

Table SI Code lists used to identify study variables

medcode	description
**Codes for polycystic ovary syndrome**	
C164.12	Stein - Leventhal syndrome
C165.00	Polycystic ovarian syndrome
7E25300	Endoscopic drilling of ovary
K591100	Oligomenorrhoea
K591200	Primary oligomenorrhoea
K591300	Secondary oligomenorrhoea
K594.00	Irregular menstrual cycle
K594z00	Irregular menstrual cycle NOS
C161000	Hypersecretion of ovarian androgen
M240.00	Alopecia
M240000	Alopecia unspecified
M240200	Male pattern alopecia
M240300	Frontal alopecia of women
M240400	Premature alopecia
M240z00	Alopecia NOS
M241.00	Hirsutism - hypertrichosis
M260.00	Acne varioliformis
M260000	Acne frontalis
M260z00	Acne varioliformis NOS
M261.00	Other acne
M261000	Acne vulgaris
M261100	Acne conglobata
M261600	Cystic acne
M261A00	Pustular acne
M261E00	Acne excoriee des jeunes filles
M261F00	Acne fulminans
M261G00	Acne agminata
M261J00	Acne necrotica
M261K00	Acne keloidalis
M261X00	Acne, unspecified
M261z00	Other acne NOS
Myu6300	[X]Other androgenic alopecia
Myu6800	[X]Other acne
Myu6F00	[X]Acne, unspecified
4473	Serum testosterone
4473100	Serum testosterone level abnormal
4474	Free androgen index
4474100	Free androgenic index abnormal
447G.00	Plasma testosterone level
447H.00	Androgen level
4Q26.00	Dihydrotestosterone level

4Q2E.00	Free testosterone level
4Q2F.00	Calculated free testosterone
ZRBs.00	Ferriman and Galwey score
K53..11	Ovarian cysts
K532.00	Other ovarian cysts
K532z00	Ovarian cyst NOS
Kyu9500	[X]Other and unspecified ovarian cysts
**Codes for diseases for exclusion**	
B540.00	Malignant neoplasm of adrenal gland
B540000	Malignant neoplasm of adrenal cortex
B540100	Malignant neoplasm of adrenal medulla
B540z00	Malignant neoplasm of adrenal gland NOS
B587.00	Secondary malignant neoplasm of adrenal gland
B7H0.00	Benign neoplasm of adrenal gland
B8yy100	Carcinoma in situ of adrenal gland
B922.00	Neoplasm of uncertain behaviour of adrenal gland
BB5h.00	[M]Adrenal cortical tumours
BB5h000	[M]Adrenal cortical adenoma NOS
BB5h100	[M]Adrenal cortical carcinoma
BB5h300	[M]Adrenal cortical adenoma, heavily pigmented variant
BB5h400	[M]Adrenal cortical adenoma, clear cell type
BB5h500	[M]Adrenal cortical adenoma, glomerulosa cell type
BB5h600	[M]Adrenal cortical adenoma, mixed cell type
BB5hz00	[M]Adrenal cortical tumours NOS
BBCF.00	[M]Adrenal rest tumour
BBD7.00	[M]Extra-adrenal paraganglioma, NOS
C15..00	Disorders of adrenal glands
C153.00	Other corticoadrenal overactivity
C155000	Adrenal medullary insufficiency
C15y.00	Other specified adrenal disorders
C15yz00	Other specified adrenal disorder NOS
C15z.00	Adrenal gland disorder NOS
Cyu4A00	[X]Other specified disorders of adrenal gland
PK1..00	Anomalies of adrenal gland
PK10.00	Aberrant adrenal gland
PK12.00	Accessory adrenal gland
PK13.00	Hypoplasia of adrenal gland
PK14.00	Ectopic adrenal gland
PK1y.00	Other specified anomalies of adrenal gland
PK1y000	Congenital cyst of adrenal gland
PK1yz00	Other congenital anomaly of adrenal gland NOS
PK1z.00	Anomalies of adrenal gland NOS
2226.11	O/E - cushingoid facies
C150.00	Cushing's syndrome
C150000	Idiopathic Cushing's syndrome
C150100	Iatrogenic Cushing's syndrome
C150111	Drug-induced Cushings syndrome

C150200	Pituitary dependent Cushing's syndrome
C150300	Ectopic ACTH secretion causing Cushing's syndrome
C150500	Alcohol-induced pseudo-Cushing's syndrome
C150z00	Cushing's syndrome NOS
Cyu4500	[X]Other Cushing's syndrome
F395100	Myopathy due to Cushing's syndrome
C150400	Nelson's syndrome
BB5y400	[M]Prolactinoma
B542.00	Malignant neoplasm pituitary gland and craniopharyngeal duct
B542000	Malignant neoplasm of pituitary gland
B542z00	Malig neop pituitary gland or craniopharyngeal duct NOS
B7H2.00	Benign neoplasm of pituitary gland and craniopharyngeal duct
B7H2.11	Pituitary adenoma
B7H2000	Benign neoplasm of pituitary gland
B7H2z00	Benign neoplasm of pituitary and craniopharyngeal duct NOS
B8yy300	Carcinoma in situ of pituitary gland
B920.00	Neop uncertain behaviour pituitary and craniopharyngeal duct
B920000	Neoplasm of uncertain behaviour of pituitary gland
B920z00	Neop uncertain behaviour pituitary and craniopharyngeal NOS
BB5V.00	[M]Pituitary adenomas and carcinomas
BB5Vz00	[M]Pituitary adenoma or carcinoma NOS
C13..00	Disorders of pituitary gland and its hypothalamic control
C131.00	Other anterior pituitary hyperfunction
C134.00	Other anterior pituitary disorder
C134z00	Other anterior pituitary disorder NOS
C134z11	Anterior pituitary hormone deficiency NEC
C137.00	Iatrogenic pituitary disorders
C137z00	Iatrogenic pituitary disorder NOS
C13z.00	Pituitary disorders NOS
Cyu4400	[X]Other disorders of pituitary gland
Cyu4M00	[X]Hyperfunction of pituitary gland, unspecified
K5B1.00	Female infertility of pituitary - hypothalamic origin
K5B1000	Primary pituitary - hypothalamic infertility
K5B1z00	Female infertility of pituitary - hypothalamic cause NOS
PK24.00	Anomalies of pituitary gland
PK24000	Aberrant pituitary gland
PK24z00	Anomaly of pituitary gland NOS

Table SII Number and percentage of PCOS women prescribed relevant drugs for PCOS both prior to and following the diagnosis of PCOS stratified by case definition

Types of drugs No. (%)	Before		After					
			2004-2007		2008-2011		2012-2014	
	Diagnosed	Probable	Diagnosed	Probable	Diagnosed	Probable	Diagnosed	Probable
Combined oral contraceptives (COC)	5136 (31.35)	7213 (50.37)	601 (16.68)	291 (17.19)	611 (20.57)	311 (16.51)	290 (20.52)	179 (14.90)
Progestin oral contraceptives (POC)	2067 (12.62)	3739 (26.11)	260 (5.48)	212 (8.11)	377 (9.18)	312 (11.58)	163 (8.29)	115 (7.24)
Intrauterine devices (IUDs)	191 (1.17)	537 (3.75)	21 (0.40)	23 (0.69)	31 (0.64)	33 (0.88)	13 (0.51)	13 (0.54)
Clomiphene	332 (2.03)	186 (1.30)	164 (3.18)	45 (1.34)	129 (2.69)	38 (0.98)	61 (2.41)	10 (0.41)
Metformin	1084 (6.62)	194 (1.35)	1125 (23.27)	88 (2.62)	991 (21.87)	112 (2.92)	459 (19.44)	51 (2.11)
Gonadotrophins	126 (0.77)	309 (2.16)	17 (0.32)	12 (0.36)	7 (0.14)	6 (0.16)	5 (0.20)	8 (0.33)
Spironolactone	174 (1.06)	61 (0.43)	101 (1.94)	9 (0.26)	101 (2.08)	10 (0.26)	37 (1.45)	7 (0.28)
Cyproterone	72 (0.44)	19 (0.13)	27 (0.51)	4 (0.12)	17 (0.35)	2 (0.05)	2 (0.08)	1 (0.04)
Flutamide	6 (0.04)	0	4 (0.08)	0	0	1 (0.03)	0	0
Eflornithine	415 (2.53)	135 (0.94)	279 (5.33)	73 (2.14)	292 (6.22)	112 (2.93)	129 (5.27)	47 (1.94)
Weight control/loss drugs	849 (5.18)	481 (3.36)	320 (6.39)	84 (2.53)	284 (6.22)	82 (2.21)	72 (3.02)	23 (0.98)
Lipid regulators	93 (0.57)	101 (0.71)	48 (0.91)	22 (0.65)	25 (0.52)	21 (0.55)	5 (0.20)	3 (0.12)
Acne-related drugs	3547 (21.64)	5453 (38.08)	600 (14.99)	585 (28.63)	583 (15.95)	664 (28.53)	226 (12.67)	373 (25.92)

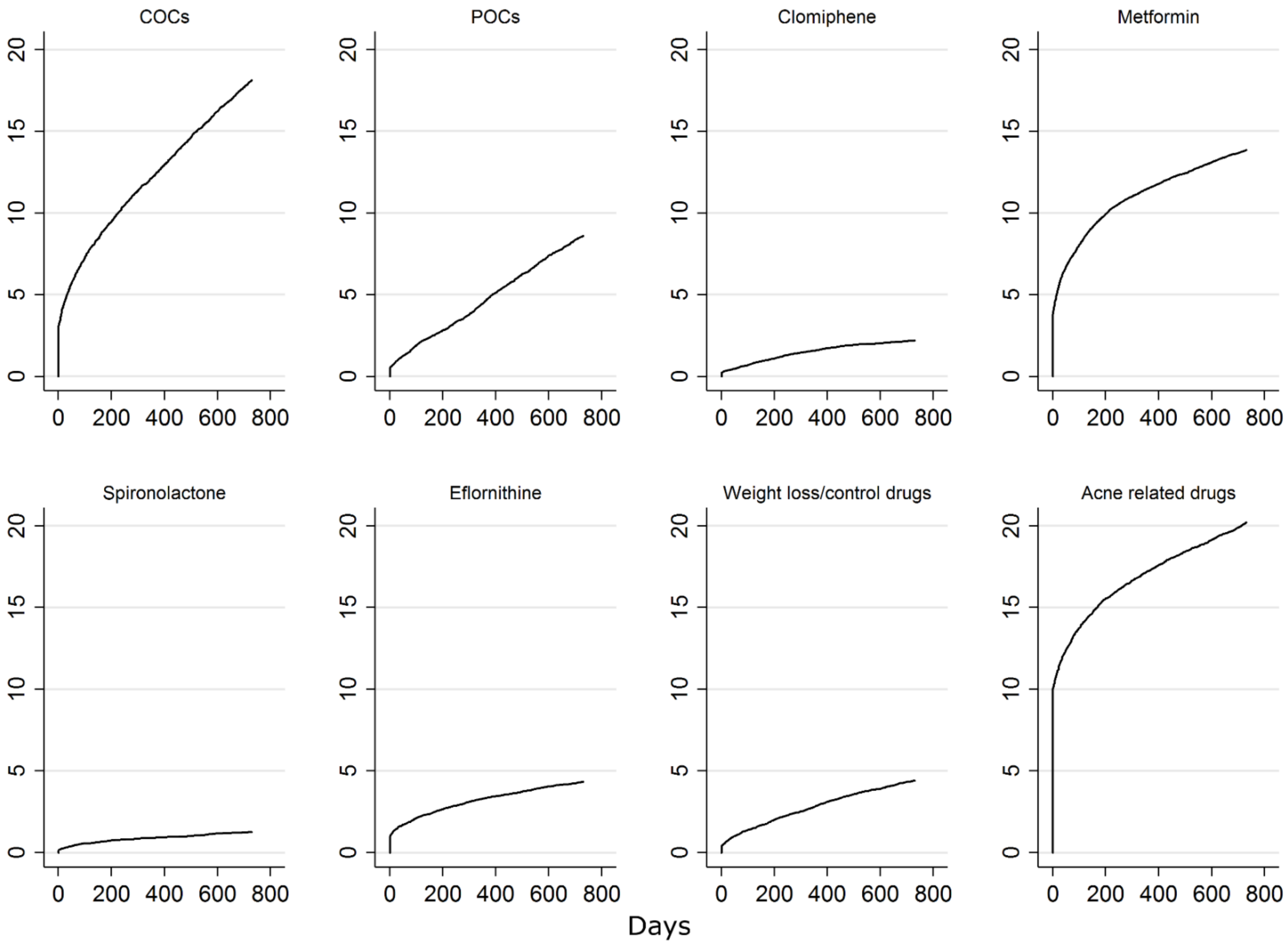

Figure SI. Plots describing the cumulative incidence of women with a prescription for each drug type over the 730 days following their index date. Results shown for the eight most commonly prescribed drugs.
